# Role of nurses in the initiation and the monitoring of Lithium

**DOI:** 10.1192/j.eurpsy.2022.470

**Published:** 2022-09-01

**Authors:** M. Moalla, A. Larnaout, D. Skhiri, R. Lansari, N. Staali, W. Melki

**Affiliations:** Razi Hospital, Psychiatry D, Manouba, Tunisia

**Keywords:** monitoring, Nurses, initiation, Lithium

## Abstract

**Introduction:**

Lithium is the oldest known treatment of bipolar disorders and remains the gold standard. Nevertheless, it remains difficult to handle, largely due to its narrow therapeutic index and its long-term side effects. Thus, it requires special initiation and monitoring measures.

**Objectives:**

This study aims to assess nurses’ knowledge and attitudes regarding lithium. A protocol on Lithium initiation and monitoring will be established.

**Methods:**

This is a descriptive study including 20 nurses in a psychiatry department conducted from January to May 2021 based on an self-assessment questionnaire that was established to assess nurses’ knowledge about Lithium, its side effects, initiation and monitoring.

**Results:**

None of the recruited nurses had any training regarding the use of lithium. The vast majority of subject (85%) said that lithium’s dosage must be individualized and adaptable to each patient throughout a specific blood test. 90% recognized renal failure as the most common contraindication of lithium. Complete Blood Count (CBC), and renal check-up were the only tests recognized as necessary by all the sample subjects. 90% answered that lithium is toxic and 65% answered that it is fatal. In case of toxicity by lithium all subjects (100%) agreed to call the responsible doctor of the patient, 25% of them chose it as a unique measure and 75% thought it was necessary to stop the lithium immediately as well.

**Conclusions:**

Lithium is considered as a double-edged sword largely due to its narrow therapeutic index. Nevertheless, nurses are undertrained when it comes to its use and manipulation.

**Disclosure:**

No significant relationships.

